# Acute Normovolemic Hemodilution Changes the Aquaporin Expression Profile in Specific Tissues and Induces Apoptotic and Inflammatory Processes in a Rat Model

**DOI:** 10.3390/medicina61091506

**Published:** 2025-08-22

**Authors:** Kerem Erkalp, Serdar Demirgan, Aslıhan Şengelen, Duygu Sultan Oran, İrem Öğütcü, Ceren Gencel-Güler, Sezin Erkalp, Ebru Burcu Demirgan, Sezen Kumaş-Solak, Nermin Yelmen, Evren Önay-Uçar

**Affiliations:** 1 Department of Anesthesiology and Reanimation, Institute of Cardiology, Istanbul University-Cerrahpaşa, 34098 Istanbul, Türkiye; 2 Department of Physiology, Cerrahpaşa Faculty of Medicine, Istanbul University-Cerrahpaşa, 34098 Istanbul, Türkiye; nermink@iuc.edu.tr; 3 Clinic of Anesthesiology and Reanimation, University of Health Sciences, Bağcılar Training and Research Hospital, 34200 Istanbul, Türkiye; serdardemirgan@hotmail.com (S.D.); sezenkumassolak@gmail.com (S.K.-S.); 4 Department of Molecular Biology and Genetics, Institute of Graduate Studies in Sciences, Istanbul University, 34116 Istanbul, Türkiye; iremogutcuu@gmail.com (İ.Ö.); cerengencel05@gmail.com (C.G.-G.); 5 Department of Molecular Biology and Genetics, Faculty of Science, Istanbul University, 34134 Istanbul, Türkiye; evrenonay@istanbul.edu.tr; 6 Experimental Research and Skills Development Center, University of Health Sciences, Bağcılar Training and Research Hospital, 34200 Istanbul, Türkiye; duygusultancelik@gmail.com; 7 School of Medicine, Koç University, 34450 Istanbul, Türkiye; sezinerkalp@hotmail.com; 8 Department of Pediatric Nephrology, University of Health Sciences, Bakırkoy Sadi Konuk Training and Research Hospital, 34200 Istanbul, Türkiye; ebruburcudemirgan@gmail.com

**Keywords:** hemodilution, fluid therapy, crystalloid solutions, aquaporins (AQPs), apoptotic and inflammatory responses

## Abstract

*Background and Objectives:* Acute normovolemic hemodilution (ANH) is commonly used to minimize perioperative blood loss and transfusion requirements. While it is considered safe, the molecular effects of ANH on vital organs remain unclear. Aquaporins (AQPs), the principal cellular water transporters, may play a role in tissue adaptation or injury under hemodilution stress. This study aimed to evaluate the impact of ANH on AQP1, AQP3, and AQP4 expression profiles and their association with apoptotic and inflammatory markers in the aorta, heart, kidney, and liver. *Materials and Methods:* Male Hannover–Sprague Dawley rats (6 months old) were assigned to control (no procedure), sham (anesthesia only), and hemodilution (anesthesia and ANH) groups. ANH was induced using balanced crystalloid infusion. Physiological parameters, blood gases, electrolytes, and metabolic profiles were monitored. At 24 h post-ANH, tissues were harvested for immunoblot analysis of AQPs, as well as apoptotic and inflammatory markers. *Results:* At 24 h post-ANH, changes in potassium, calcium, and glucose levels, decreased hematocrit, increased lactate, decreased pH, base excess, and PaCO_2_ were detected, indicating mild metabolic acidosis due to tissue hypoxia and impaired oxygen delivery. Apoptotic and inflammatory responses were observed across all tissues, but AQP alterations were organ-specific. In the heart, AQP1 downregulation correlated inversely with NF-κB and TNF-α levels, while AQP3 upregulation positively correlated with apoptosis. The aorta showed the opposite pattern. In the kidney, AQP4 downregulation was strongly associated with apoptosis and inflammation. Furthermore, ANH selectively increased the AQP3 expression without affecting AQP1 or AQP4 in the liver. *Conclusion:* ANH induces differential aquaporin expression patterns in major organs, with tissue-specific associations with apoptosis and inflammation. These findings highlight a potential mechanistic role for AQPs, particularly AQP1 and AQP3, in modulating tissue response to hemodilution. These molecular adaptations may serve as early indicators of tissue stress, suggesting clinical relevance for perioperative fluid strategies.

## 1. Introduction

Hemodilution is a critical procedure used for surgical applications with a high risk of blood loss. It involves collecting blood into collection bags during surgery and simultaneously giving the patient crystalloids or colloids, diluting normal blood components [1,2]. Four different types of hemodilution were described in previous years: (1) Normovolemic hemodilution, which occurs due to spontaneous transcapillary blood volume after bleeding of 10–15% of the blood volume. (2) Hypovolemic hemodilution, which occurs due to more severe blood losses and inadequate capillary refill, but remains stable. (3) The acute normovolemic hemodilution technique, which helps reduce red blood cell concentrations by immediately replacing blood volume with cell-free plasma-like fluids in cases of acute blood loss, thereby maintaining blood volume within normal ranges. (4) Acute hypervolemic hemodilution, which occurs due to intravenous fluid resuscitation with colloids and cell-free solutions after hemorrhagic shock [3]. Hemodilution occurs due to both changes in the ionic balance of the intravascular environment (increase or decrease) and changes in the fluidity of the blood in intravenous fluid therapy (IFT) [4].

Acute normovolemic hemodilution (ANH) is a strategy used to reduce the need for blood transfusion. The first step in ANH is the acute and controlled removal of whole blood, and then the blood loss is replaced with either the same volume of blood or colloid or with crystalloid at three times the volume of blood lost [5]. Since its description in the 1970s, ANH has been used in surgical procedures to reduce the need for allogeneic blood transfusion. ANH may be a critical alternative to preoperative autologous donation because it does not require additional patient visits for blood donation before surgery [6]. ANH is generally deemed a cheap, safe, and broadly available technique but carries potential side effects and risks. Its positive effects include reducing the need for red blood cell (RBC) transfusions, decreasing the likelihood of transfusion-related adverse reactions, improving the coagulation profile after cardiopulmonary bypass (CPB), reducing both the activation of inflammatory pathways and the consumption of clotting factors and platelets, decreasing blood viscosity, and enhancing capillary perfusion and microcirculation. Its side effects include coagulopathy, hypoxia, electrolyte imbalances, impaired immune function, cardiovascular strain, and decreased intravascular osmotic pressure, which can cause edema [7,8]. The potential adverse effects of ANH and the affected cellular and molecular mechanisms remain the subject of ongoing research.

Osmoregulation and body water homeostasis depend on regulating aquaporin (AQP) protein family members, which are essential cell water transporters. They enable water transport and the passage of small molecules, such as glycerol, through biological channels in many epithelial and endothelial cells [9]. Notably, AQPs play a regulatory role in forming and resolving edema. However, what is known about this complex dual effect is quite limited [10]. Furthermore, there is insufficient information regarding the roles of AQPs in hemodilution processes. An experimental porcine model study emphasized the association between fluid resuscitation and the formation of brain damage in hemorrhagic shock. Fluid resuscitation using colloids, particularly gelatin–polysaccharide, compared to hydroxyethyl starch and balanced electrolyte solution, increased AQP4 levels and was associated with cerebral injury [11]. Some reports also suggest that AQPs, due to their presence in the endothelial membrane, may cause hemodilution by increasing plasma transport. A high amount of AQP1 in the fetal endothelial membrane may be associated with filtration in the fetal anemia model, when induced by isovolumetric hemorrhage in ewes [12]. In another study, AQP1 levels were examined in newborn lamb models that replicated infant CPB with hypothermic circulatory arrest, and an increase in AQP1 mRNA levels was detected, suggesting a possible relationship with pulmonary edema [13]. On the other hand, AQP1 and AQP3 have been shown not to affect H_2_O_2_ permeability in red blood cells [14].

Beyond their physiological roles, AQPs have been increasingly gaining attention within surgical and critical care settings due to their roles in fluid homeostasis, edema control, and tissue-specific responses to hemodynamic stress. Studies utilizing experimental models of hemorrhagic shock, cardiopulmonary bypass, and fluid resuscitation have revealed altered AQP expression, notably an upregulation of AQP4 in cerebral edema and modulation of AQP1 in pulmonary injury [15,16,17]. Such evidence highlights the relevance of elucidating AQP regulation during acute fluid management, particularly in major surgery or intensive care environments, where rapid shifts in fluid and hemodynamics are frequent. Dysregulated AQP expression may contribute not only to edema formation but also to impaired barrier function and organ dysfunction, ultimately affecting clinical outcomes [18,19]. Therefore, investigating AQPs within the context of ANH provides a valuable translational framework to explore early cellular stress responses and could inform perioperative fluid strategies aimed at reducing organ injury.

Considering the widespread clinical application of ANH in surgical settings, it is plausible that ANH may induce organ-specific molecular alterations, including changes in AQP expression patterns, which could reflect early cellular stress responses. Herein, we hypothesized that ANH would cause tissue-specific changes in AQP expression linked to early adaptive or stress responses and molecular injury markers, and investigated the short-term effects of ANH on AQP1, AQP3, and AQP4 protein expression in the heart, aorta, kidney, and liver, as well as its impact on systemic hemodynamics, blood gas levels, and markers of apoptosis and inflammation. This study is the first to link tissue-specific AQP expression with apoptotic and inflammatory responses in the rat ANH model, showing that ANH leads to early systemic and tissue-specific alterations in these parameters.

## 2. Materials and Methods

### 2.1. Ethics Statement and Animals

Eighteen male juvenile (6 months of age) Hannover–Sprague Dawley (Han:SPRD) rats (400–500 g) were included in this study. The animals were kept in a controlled environment with a 12 h light/dark cycle, 60% humidity, and a temperature of 20–24 °C; free access to food and water was allowed. Ethical approval was provided by the Animal Ethics Committee of University of Health Sciences, Bağcılar Training and Research Hospital, Istanbul, Türkiye (approval protocol number: HADYEK-2023/29; 24 April 2023). All animal care and experiments were conducted at Istanbul Bağcılar Training and Research Hospital Experimental Research and Skill Development Training Centre (BADABEM) by ARRIVE guidelines and the National Research Council’s Guide for the Care and Use of Laboratory Animals. All efforts were made to minimize animal suffering.

### 2.2. Experimental Setup and Design

The groups were determined by randomizing (generated using the standard = RAND function in Microsoft Excel) the experimental model and the controls as follows: ***(1) Control group***: no procedure was performed (Group-C, n = 6). ***(2) Sham control group***: only anesthesia was applied (Group-S, n = 6). ***(3) Experimental hemodilution group***: anesthesia and acute normovolemic hemodilution (ANH) were applied (Group-ANH, n = 6).

**Figure 1:**
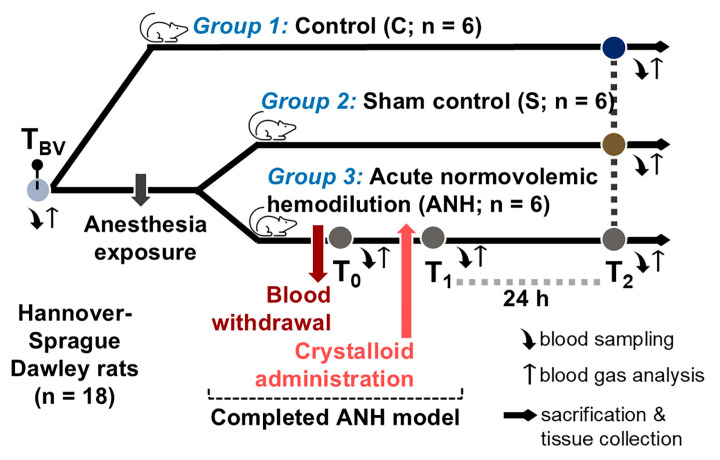
Outline of the experimental protocol. Juvenile male Hannover–Sprague Dawley rats were randomly assigned to three groups (n = 6/group): Group-1 (control, C), no procedure was performed. Group-2 (sham control, S), only anesthesia (2% sevoflurane in 50–50% oxygen–air for 1 h) was applied. Group-3 (experimental hemodilution), anesthesia and acute normovolemic hemodilution (ANH) were applied. To achieve the ANH, the allowable blood volume was withdrawn from the left femoral artery (T_0_ time point) and 1:3 volume balanced crystalloid solution was administered (i.v., T_1_ time point). Blood samples were collected at T_BV_ (for baseline parameters), T_0_, T_1_ (during hemodilution), and T_2_ (post-operative day 1) time points for further analysis. After euthanasia with deep anesthesia, aorta, heart, liver, and kidney tissues were collected for immunoblotting analysis.

Before the procedure, the rats’ body weights were noted, and the estimated blood volume (EBV) was calculated based on those weights. The EBV in rats is 64 mL/kg [20]; this value corresponds to ~5–7 mL/100 g [21]. In all groups, one mL of blood sample was collected before and after the protocol. Baseline values (BVs) were checked before starting the experiments in all rats. Inhalation anesthesia was applied to the rats in Group-S and Group-ANH by administering 2% sevoflurane (in 50–50% oxygen–air for 1 h). Furthermore, in the ANH group, blood samples were collected at T_0_ and T_1_ time points of hemodilution process. Subsequently, rats were housed individually in cages. To monitor the postoperative effect, blood samples were collected by cardiac puncture 24 h after the hemodilution model (T_2_ time point: first postoperative day). In the control or sham groups, procedures associated with the ANH protocol, including blood withdrawal and crystalloid infusion, were not performed; only small-volume (1 mL) blood samples were collected at T_BV_ and T_2_ time points for the purpose of blood parameter comparison. After euthanasia with deep anesthesia by i.v. injection of ketamine, aorta, heart, liver and kidney tissues were collected for immunoblotting analysis. The schematic representation of the experimental design is presented in Figure 1.

### 2.3. Experimental Acute Normovolemic Hemodilution Model

The experimental ANH model was processed during the maintenance of general anesthesia. Therefore, rats in Group-S and Group-ANH were anesthetized with 2% sevoflurane (Sojourn^®^ inhalation solution 100%, Adeka, Istanbul, Türkiye) in 50–50% oxygen–air. Normothermia (37 °C ± 1 °C) was preserved with heating pads.

Rats were placed in supine position, and an intravenous catheter-26G was placed in the tail vein and left femoral artery. To achieve the rodent model of acute normovolemic hemodilution, the allowable blood volume was withdrawn from the left femoral artery in Group-ANH rats (T_0_ time point blood samples) and blood samples were collected for the target hematocrit (HCT). The allowable blood volume was calculated using the following formula [6]: Estimated blood volume (mL) × [(HCT_current_ − HCT_target_)/HCT_average_]. The volume of blood collected for hemodilution was ~1/3 of the total blood volume in adult rats. Similarly, for a 70 kg adult human with an estimated blood volume of 5 L, collecting four units of blood (~450 mL per unit, totaling 1.8 L) also constitutes roughly 1/3 of total blood volume, thereby resulting in profound hemodilution. This experimental model represents deep hemodilution strategies, rarely applied in clinical practice [22,23,24], and aligns with observed reductions in hematocrit reported in other deep hemodilution studies [25,26]. This deep hemodilution model was selected to facilitate the investigation of early molecular responses. To maintain normovolemia, 1:3 volume balanced crystalloid solution (3 mL, i.v. injection, Polifleks-Izolen, Polifarma, Istanbul, Türkiye) was administered instead of 1 mL of lost blood volume via the intravenous catheter in the tail [2,6]. The replacement fluid used (Polifleks-Izolen) is a balanced crystalloid containing sodium (140 mmol/L), potassium (10 mmol/L), calcium (2.4 mmol/L), magnesium (1.5 mmol/L), chloride (103 mmol/L), acetate (47 mmol/L), and citrate (2.55 mmol/L). The hemodilution process was completed in approximately 30 min (blood withdrawal lasted 10 min, crystalloid administration took 20 min). Finally, 1 mL blood sample was withdrawn (T_1_ time point blood samples) for blood gas analysis after intravenous fluid replacement.

### 2.4. Hemodynamic and Blood (Gas) Measurements

Throughout the procedure, all animals were monitored for respiratory rate (RR, bpm), oxygen saturation (SpO_2_, %), and heart rate (HR, bpm) using a low-noise cabled pulse oximetry sensor (placed on the right hind leg of rats; Masimo SET Masimo-LNCS; Irvine, CA, USA) and an oximeter (Masimo, Rad-97^®^ Pulse Oximeter, Irvine, CA, USA). The SpO_2_ and HR were set at 2 s intervals and maximal sensitivity. Also, body temperature was monitored continuously. Arterial blood gas analysis for hematocrit (HCT) value (%), calculated serum osmolality (mOsmC) (mmoL/kg), pH value, partial carbon dioxide pressure (PaCO_2_, mmHg), standard base excess (BE, mmol/L), lactate levels (mmol/L), and glucose (mg/dL) were obtained using an ABL90 FLEX PLUS blood gas analyzer (Radiometer, Copenhagen, Denmark). Additionally, concentrations (mmol/L) of sodium (Na^+^), potassium (K^+^), calcium (Ca^2+^), and chlorine (Cl^−^) were measured using ion-selective electrode (ISE) technology integrated into the same blood gas analyzer.

### 2.5. Protein Extraction from Tissues and Western Blot Analysis

As previously described by Demirgan et al. [27] and Yayla-Tunçer et al. [28], protein extraction from tissues and Western blot analysis were performed. For aorta tissues, each sample was frozen in liquid nitrogen, pulverized with a mortar/pestle, and homogenized in TRIzol Reagent (500 μL) according to the manufacturer’s instructions (Invitrogen, Carlsbad, CA, USA). Furthermore, isolation from heart, liver, and kidney tissues was performed using pre-chilled lysis buffer (50 mM Tris-HCl [pH 7.6], 150 mM NaCl, 1% NP-40, 1% Triton X-100, 10% glycerol, 1 mM PMSF, and EDTA-free PIC) after the samples were frozen in liquid nitrogen in a mortar and pulverized. All lysates obtained from organs were precipitated using the methanol–chloroform precipitation method [29]. Next, protein concentration was determined by BCA commercial kit (iNtRON-Biotechnology, Seongnam, Republic of Korea). Equal quantities of proteins (30 µg/well) were separated under reducing conditions by SDS-PAGE, and transferred to PVDF membrane (Bio-Rad, Hercules, CA, USA). After blocking with non-fat dry milk (5%, prepared in TBST), the membrane was incubated with the primary antibody (overnight at 4 °C; Table 1), washed with TBST, incubated with the secondary antibody (2 h at RT; Table 1), and washed again. β-actin was used for loading control (see supplementary Figure S1 for all reference protein images). Immunoblots were determined using enhanced chemiluminescence (ECL) plus substrate kit (Thermo, Kwartsweg, The Netherlands), imaged using a ChemiDoc XRS system (Bio-Rad), and quantified using ImageLab 6.0.1 software (Bio-Rad).

### 2.6. Statistical Analysis

All quantitative data were presented as mean ± SD. Statistical analyses were performed on GraphPad Prism statistical software v10.3.0 (San Diego, CA, USA). For the normality check, the Shapiro–Wilk test was used. The Student’s *t*-test was utilized to evaluate the difference between the two groups. A one/two-way analysis of variance (ANOVA) followed by Tukey post hoc test was performed for multiple comparisons. The strength of relationship between two variables was tested using Spearman’s correlation (r values for correlation: 0.20–0.39 for “weak”, 0.40–0.59 for “moderate”, 0.60–0.79 for “strong”, and 0.80–1.0 for “very strong”). The criterion for statistical significance was *p* < 0.05.

## 3. Results

### 3.1. Bioparameters, Acid Base Status, and Metabolic Parameters

The animals were closely monitored throughout the experimental protocol. All animals in the ANH group survived the hemodilution period. During the entire experimental period, respiratory rate (RR), oxygen saturation (SpO_2_), heart rate (HR, bpm), and temperature were continuously recorded; RR, SpO_2_, and HR values were virtually identical in the three groups, and the average body temperature of the animals was 37.5 ± 0.45 °C. As indicated in Table 2, there was no difference between the body weights of the animals (*p* > 0.05). The average blood taken from the ANH group rats was 11.67 ± 0.83 mL. No significant intergroup differences were detected in most blood electrolytes and metabolites (Table 2). However, trends in the ANH group suggest early physiological disturbance. Mild increases in sodium and calcium levels, along with decreases in glucose levels, were observed 24 h post-ANH, although these changes were not statistically significant. A considerable elevation in potassium at T_2_ in the ANH group was noted (*p* = 0.0121).

**Figure 2:**
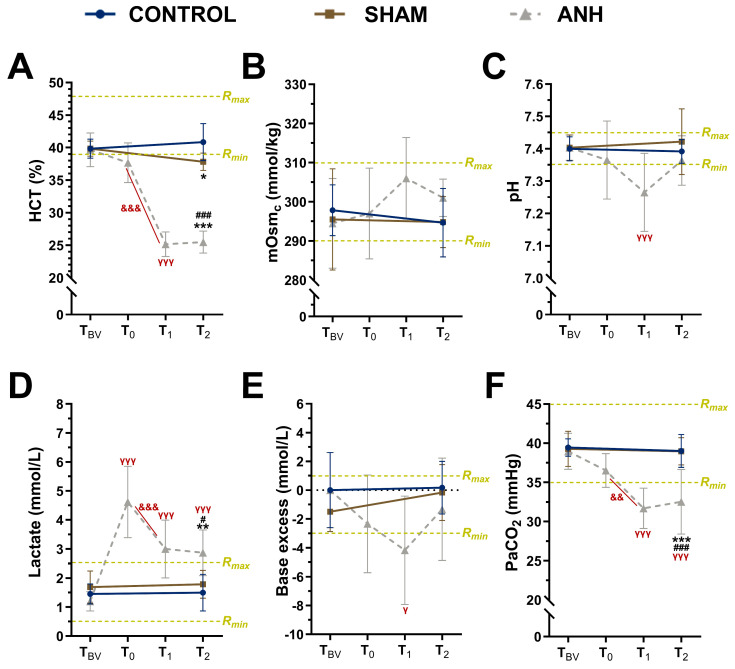
Changes in arterial blood gas parameters from T_BV_ to T_2_. (**A**) hematocrit (HCT) value (%), (**B**) serum osmolality (mOsmc, mmol/kg), (**C**) pH, (**D**) lactate (mmol/L), (**E**) base excess (mmol/L), and (**F**) partial carbon dioxide pressure (PaCO_2_, mmHg) values of control, sham, and ANH (acute normovolemic hemodilution) groups. Yellow horizontal lines show the minimum (R_min_) and maximum (R_max_) reference values for the measured parameters in rats. Data are presented as mean ± standard deviation (SD). * *p* < 0.05, ** *p* < 0.01, and *** *p* < 0.001 compared to control group; # *p* < 0.05 and ### *p* < 0.001 compared to sham group; γ *p* < 0.05 and γγγ *p* < 0.001 indicate comparison between T_BV_ and other ANH time points. && *p* < 0.01 and &&& *p* < 0.001 indicate time effect in the ANH group.

As shown in Figure 2A, a significant reduction in HCT was detected in the ANH group between the T_BV_ and T_1_ time points (*p* < 0.0001), and compared to the sham and control groups (according to T_2_, *p* < 0.0001), confirming the ANH model [30,31,32]. While serum osmolality in the control and sham animals was in the normal range, with an average of 295 mmoL/kg [33], it was found to increase to >300 mmoL/kg at the T_1_ time point after the application of crystalloid solution in the ANH group. As shown in Figure 2C,E, a downward trend in arterial pH and base excess was observed in the ANH group following hemodilution, suggesting the development of mild metabolic acidosis. Specifically, arterial pH significantly decreased between the T_BV_ and T_1_ time points in the ANH group (*p* < 0.0001), while base excess also showed a significant reduction during the same interval (*p* = 0.0317). These findings reflect acute acid–base disturbances triggered by the rapid volume shift, even though the overall group comparisons did not reach statistical significance. Due to hemodilution, the blood lactate concentration in the ANH group increased markedly at the T_1_ time point (*p* < 0.0004), likely due to hypoperfusion-induced anaerobic metabolism, and remained elevated at the T_2_ time point compared to the control (*p* = 0.0018) and sham (*p* = 0.0157) groups (Figure 2D). Additionally, a within-group analysis of the ANH group showed that the lactate levels at T_0_ (*p* < 0.0001), T_1_ (*p* < 0.0001), and T_2_ (*p* = 0.0003) time points were all significantly higher compared to the T_BV_, indicating a persistent metabolic response throughout the experimental timeline. The PaCO_2_ also decreased in the ANH group in a time-dependent manner (*p* = 0.0023), and the reduction was significant compared to the sham and control groups at T_2_ (*p* < 0.0001, Figure 2F). When compared with the baseline values, the PaCO_2_ levels in the ANH group were lower at both the T_1_ (*p* < 0.0001) and T_2_ (*p* < 0.0001) time points.

### 3.2. The Effects of Acute Normovolemic Hemodilution on Aquaporin Expression Profile in Different Tissues

**Figure 3:**
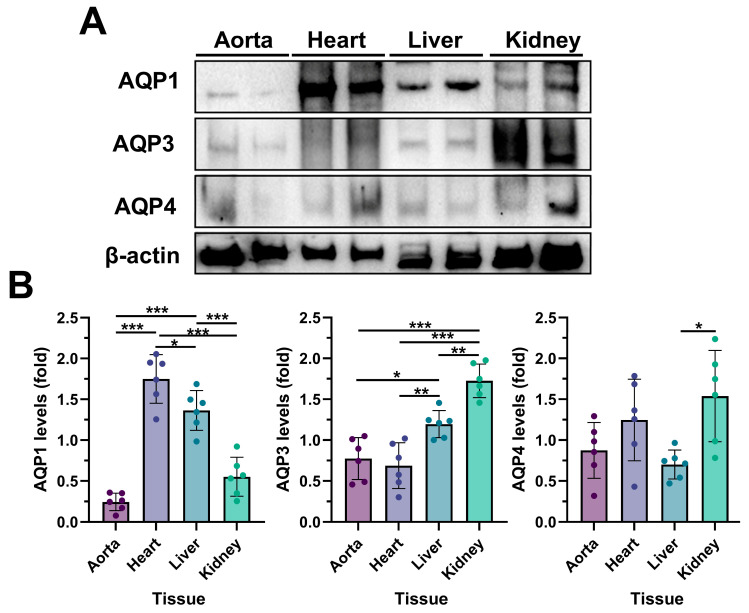
Aquaporins (AQPs) are expressed differently in different tissues. (**A**) Western blot analysis of AQP1, AQP3, and AQP4 in the aorta, heart, liver, and kidney (β-actin as a loading control). (**B**) Quantitative analysis of immunoblotting in different tissues. Data are presented as mean ± standard deviation (SD). (n = 6/group). * *p* < 0.05, ** *p* < 0.01, and *** *p* < 0.001 indicates comparison between different groups.

To identify tissue-specific expression patterns of AQP1, AQP3, and AQP4 under normal physiological conditions, independent of any intervention such as anesthesia or fluid administration, immunoblotting was performed in the aorta, heart, liver, and kidney tissues collected from the control group at the T_2_ time point (Figure 3A). As expected, AQP1, AQP3, and AQP4 expression levels were tissue-specific (Figure 3B). AQP1 level was highest in the heart. AQP3 and AQP4 expressions were highest in the kidney. Additionally, there was no significant difference in AQP3 levels between the aorta and the heart. AQP4 levels were only markedly higher in the kidney than in other tissues.

**Figure 4:**
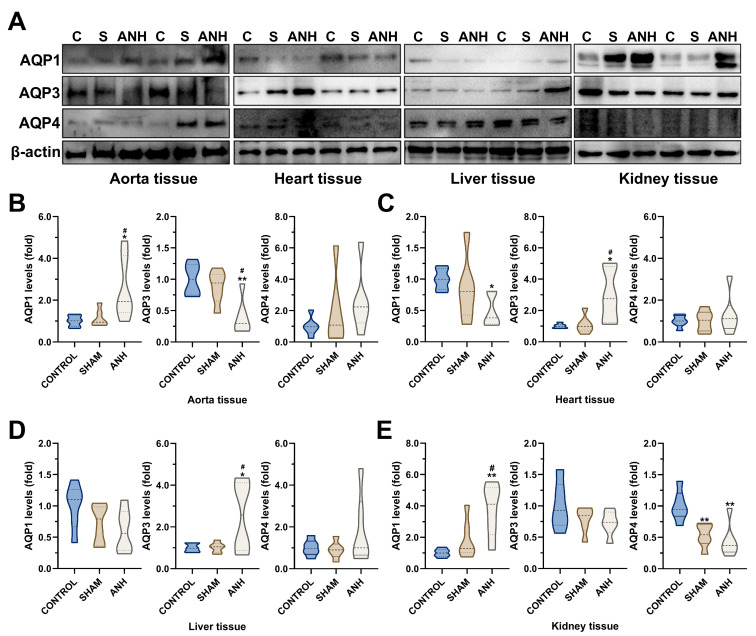
Acute normovolemic hemodilution (ANH) alters the aquaporin (AQP) expression profile in a tissue-specific manner. (**A**) Western blot analysis of AQP1, AQP3, and AQP4 in (**B**) aorta, (**C**) heart, (**D**) liver, and (**E**) kidney tissues (β-actin as a loading control). Data are presented as mean ± standard deviation (SD). * *p* < 0.05 and ** *p* < 0.01 compared to control (C) group; # *p* < 0.05 compared to sham (S) group.

ANH led to a tissue-specific change in the AQP expression profile (Figure 4A). In the aorta, the AQP1 level increased (2.52-fold change, *p* = 0.0280 and *p* = 0.0451, respectively) and the AQP3 level (2.56-fold change, *p* = 0.0032 and *p* = 0.0153, respectively) decreased statistically significantly in the ANH group compared to the control and sham groups. In contrast, no significant difference (*p* > 0.05) was observed in AQP4 level in any group (Figure 4B). In contrast to the results from the aorta samples, in the heart, the decrease in AQP1 level (1.53-fold change, *p* = 0.0472) and the increase in AQP3 level (2.94-fold change, *p* = 0.0131) were significant in the ANH group compared to the control (Figure 4C). In the liver, only the rise in AQP3 level (2.52-fold change, *p* = 0.0429 and *p* = 0.0459, respectively) in rats treated with ANH was statistically significant compared to control and sham animals (Figure 4D). In the kidney samples, the AQP1 level (3.03-fold change, *p* = 0.0036 and *p* = 0.0388, respectively) was observed to be significantly increased in the ANH group compared to the control and sham group. AQP4 expression was downregulated in the ANH group (*p* = 0.0028), but a similar reduction was detected in sham animals (*p* = 0.0099, Figure 4E).

### 3.3. The Effects of Acute Normovolemic Hemodilution on Apoptotic and Inflammatory Responses in Different Tissues

**Figure 5:**
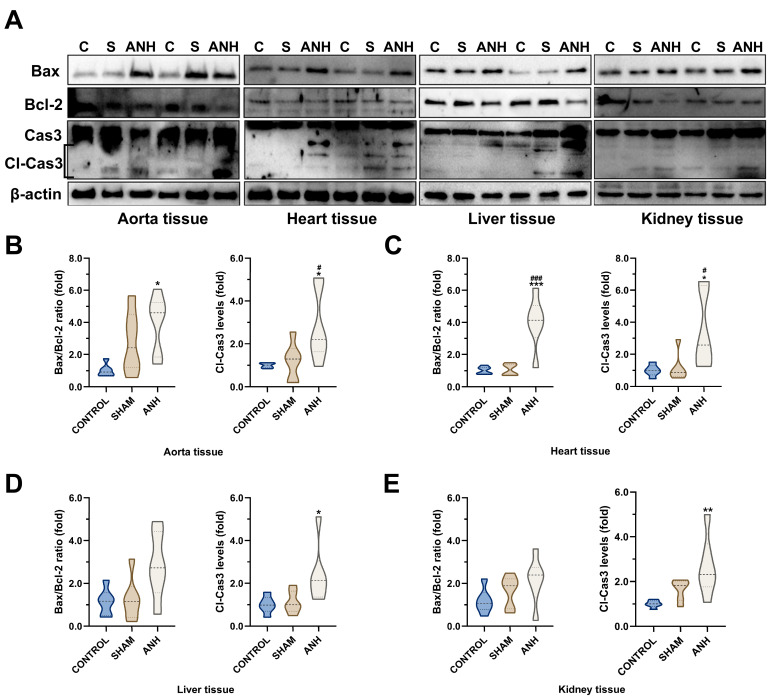
Acute normovolemic hemodilution (ANH) causes apoptotic response in different tissues. (**A**) Western blot analysis of Bax (Bcl-2-associated X protein), Blc-2 (B-cell lymphoma 2), and Cl-Cas3 (cleaved Caspase-3) in (**B**) aorta, (**C**) heart, (**D**) liver, and (**E**) kidney tissues (β-actin as a loading control). Data are presented as mean ± standard deviation (SD). * *p* < 0.05, ** *p* < 0.01, and *** *p* < 0.001 compared to control (C) group; # *p* < 0.05 and ### *p* < 0.001 compared to sham (S) group.

The effect of ANH on tissue damage was investigated by determining induced apoptotic and inflammatory responses. The apoptotic impact was revealed by examining changes in the Bax/Bcl-2 ratio and cleaved Caspase-3 levels (Figure 5A).

**Figure 6:**
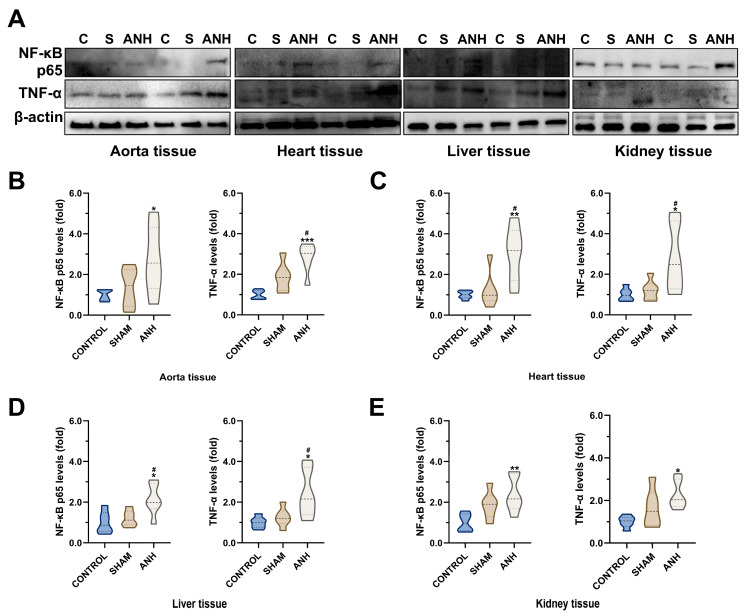
Acute normovolemic hemodilution (ANH) causes inflammatory response in different tissues. (**A**) Western blot analysis of NF-κB (nuclear factor kappa B) p65 and TNF-α (tumor necrosis factor alpha) in (**B**) aorta, (**C**) heart, (**D**) liver, and (**E**) kidney tissues (β-actin as a loading control). Data are presented as mean ± standard deviation (SD). * *p* < 0.05, ** *p* < 0.05, and *** *p* < 0.001 compared to control (C) group; # *p* < 0.05 compared to sham (S) group.

Due to the ANH process, Bax/Bcl-2 ratio and Caspase-3 fragments increased by 3.94 (*p =* 0.0136) and 2.88 (*p* = 0.0281) times, respectively, in aorta samples (Figure 5B). In the heart, Bax/Bcl-2 ratio (4-fold, *p* < 0.0002) and cleaved Caspase-3 levels (3.40-fold, *p* = 0.0409) were found to be significantly higher in the ANH group compared to control (Figure 5C). In liver tissue, the ANH group showed a marked increase in the cleaved Caspase-3 fragments (2.40-fold, *p* = 0.0405) compared to the control (Figure 5D). In the kidney, only an increase in active Caspase-3 levels (2.65-fold, *p* = 0.0091) was determined (Figure 5E). In addition, to evaluate the inflammatory response-related impact caused by ANH treatment, NF-κB p65 and TNF-α levels were analyzed by immunoblotting (Figure 6A). As shown in Figure 6B, ANH led to an increase in NF-κB p65 and TNF-α levels in the aorta (2.27-fold, *p* = 0.0417; 2.81-fold, *p* = 0.0003), heart (3.03-fold, *p* = 0.0055; 2.81-fold, *p* = 0.0187), liver (2.13-fold, *p* = 0.0121; 2.43-fold, *p* = 0.0128), and kidney (2.38-fold, *p* = 0.0086; 2.27-fold, *p* = 0.0121) compared to control (some were significant according to both control and sham).

### 3.4. Relationship Between Aquaporin Expression and Apoptotic/Inflammatory Response Markers in Experimental Rats with and Without Acute Normovolemic Hemodilution

**Figure 7:**
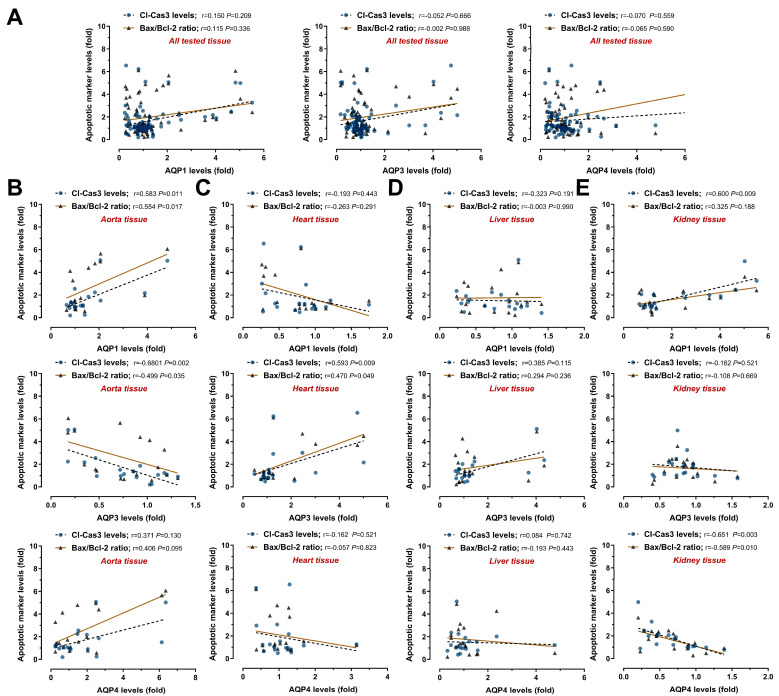
Relationship between expression levels of aquaporins (AQPs) and apoptotic markers in aorta, heart, liver, and kidney using all data from the control, sham, and ANH (acute normovolemic hemodilution) groups. Immunoblot results for all animals were used in correlation analyses, and the correlation of AQP1, AQP3, and AQP4 levels with the Bax (Bcl-2-associated X protein)/Bcl-2 (B-cell lymphoma 2) ratio and Cl-Cas3 (cleaved Caspase-3) levels was evaluated. (**A**) When the results for the four tissues were evaluated together, no correlation was observed. Notably, a tissue-specific positive or inverse correlation was detected in (**B**) aorta, (**C**) heart, (**D**) liver, and (**E**) kidney tissue samples. The strength of relationship between two variables was tested using Spearman’s correlation (r values for correlation: 0.20–0.39 for “weak”, 0.40–0.59 for “moderate”, 0.60–0.79 for “strong”, and 0.80–1.0 for “very strong”).

**Figure 8:**
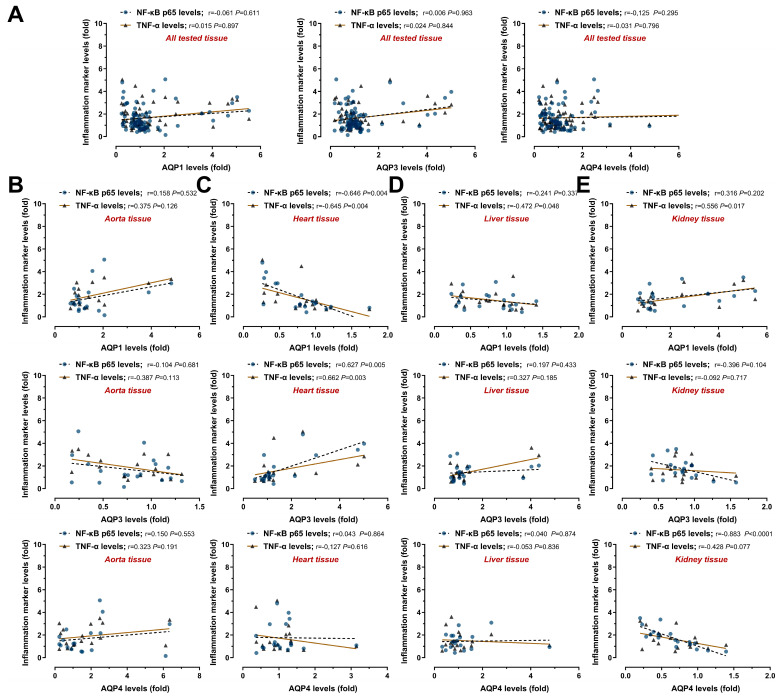
Relationship between expression levels of aquaporins (AQPs) and inflammation markers in aorta, heart, liver, and kidney using all data from the control, sham, and ANH (acute normovolemic hemodilution) groups. Immunoblot results of all animals were used in correlation analyses, and the correlation of AQP1, AQP3, and AQP4 levels with the NF-κB (nuclear factor kappa B) p65 and TNF-α (tumor necrosis factor alpha) levels was evaluated. (**A**) When the results of the four tissues were evaluated together, no correlation was observed. Notably, tissue-specific positive or inverse correlation was detected in (**B**) aorta, (**C**) heart, (**D**) liver, and (**E**) kidney tissue samples. The strength of relationship between two variables was tested using Spearman’s correlation (r values for correlation: 0.20–0.39 for “weak”, 0.40–0.59 for “moderate”, 0.60–0.79 for “strong”, and 0.80–1.0 for “very strong”).

The immunoblot results obtained from all groups were used to analyze the correlation of AQPs with apoptosis and inflammation markers. While no correlations were observed when all the tissue results were evaluated together (Figure 7A and Figure 8A), remarkable relationships were detected when each tissue was assessed separately.

In the aorta tissue samples, apoptosis markers showed a moderate positive correlation with AQP1 (Bax/Bcl-2, r = 0.554 *p* = 0.017; Cl-Cas3, r = 0.583 *p* = 0.011) and a moderate inverse correlation with AQP3 (Bax/Bcl-2, r = −0.680 *p* = 0.002; Cl-Cas3, r = 0.499 *p* = 0.035) (Figure 7B). However, there was no significant correlation with the inflammation markers (Figure 8B). In the heart tissue samples, only AQP3 showed a significant moderate positive correlation with Bax/Bcl-2 ratio (r = 0.470 *p* = 0.049) and cleaved Caspase-3 levels (r = 0.593 *p* = 0.009) (Figure 7C). NF-κB p65 and TNF-α levels were also strongly inversely correlated with AQP1 (r = −0.646 *p* = 0.004, r = −0.645 *p* = 0.004, respectively) and strongly positively correlated with AQP3 (r = 0.662 *p* = 0.005, r = 0.662 *p* = 0.003, respectively) (Figure 8C). In the liver tissue samples, there was no association between AQP levels and apoptosis (Figure 7D). A marked moderate inverse correlation was observed between AQP1 and TNF-α (r = −0.472 *p* = 0.048) (Figure 8D). In the kidney tissue samples, only AQP4 level was found to be strongly inversely correlated with Bax/Bcl-2 ratio (r = −0.589 *p* = 0.010) and active caspase-3 level (r = −0.651 *p* = 0.003) (Figure 7E). AQP4 expression also had a strong inverse correlation with NF-κB p65 level (r = −0.883 *p* < 0.0001) and a moderate inverse correlation with TNF-α level (r = −0.482 *p* = 0.077) (Figure 8E). Our correlation analysis between AQP levels and apoptotic and inflammatory responses showed tissue-specific AQP expression profiles.

## 4. Discussion

Perioperative fluid management has been a subject of debate for many years. Acute normovolemic hemodilution (ANH) is one of the prominent strategies used to prevent blood loss or hypovolemia [1,2]. Although this intervention in the perioperative setting has advantages, it has potential side effects and risks, such as coagulopathy, edema, hypoxia, endothelial damage, and acidosis [7,8]. Therefore, determining the effects of the administered fluids on tissues/organs and potential damage will be of clinical interest, particularly in high-risk surgical settings. It is noteworthy that the heart is particularly sensitive to fluid imbalances, since it has at least 10 times more fluid flow than other tissues [34,35]. Furthermore, organs such as the kidney [31,36,37], intestine [31,38], spleen [31], and brain [11,39] have limited tolerance to changes in blood oxygen content (hemoglobin concentration and hematocrit), so they are adversely affected by processes such as hemodilution. Therefore, in the present study, we investigated the effect of ANH on tissue damage in a rat model by evaluating apoptotic and inflammatory marker protein levels in the aorta, heart, kidney, and liver tissue samples. We also investigated the levels of AQP1, AQP3, and AQP4, a family of aquaporin proteins that play crucial roles in fluid flux regulation, membrane permeability, water transport, etc. [9,10], to gain insight into the roles of AQPs in hemodilution management.

Herein, we formed an experimental ANH rat model using a balanced crystalloid solution. As shown in Figure 2, the decrease in hematocrit (HCT) value at the T_1_ time point confirmed hemodilution, similar to previous reports [30,31,32]. Although our model reduced hematocrit from ~40% to ~25%, exceeding typical clinical ANH, this approach was selected to uncover early molecular responses, as demonstrated in prior experimental deep hemodilution studies [25,26]. Our model corresponds to deep hemodilution strategies, which are less frequently used in clinical practice [22,23,24]. The corresponding fluctuations observed in serum osmolality (mOsmc), arterial pH, lactate, base excess, and partial carbon dioxide pressure (PaCO_2_) further support the physiological impact of hemodilution. While the control and sham animals maintained relatively stable profiles across these parameters, the ANH group demonstrated transient but notable deviations. Specifically, the significant increase in arterial lactate levels post-hemodilution, accompanied by reductions in base excess and pH, suggests the onset of mild metabolic acidosis, likely secondary to tissue hypoxia and impaired oxygen delivery [40,41]. Lactate, as a by-product of anaerobic glycolysis, provides a sensitive indicator of cellular oxygen deficiency. The concurrent decrease in PaCO_2_ may reflect compensatory hyperventilation in response to emerging acid–base imbalances, or a shift in glucose metabolism favoring anaerobic fermentation rather than oxidative phosphorylation [31,41]. Similar metabolic alterations have been reported in experimental models of hemodilution and hypoxia, with elevated lactate levels interpreted as a reversible cellular response to acute systemic hypoxia and compromised perfusion [42,43,44]. Our findings underscore the systemic sensitivity to rapid volume shifts and emphasize the necessity of meticulous metabolic monitoring during fluid-based hemodilution strategies. Although not directly examined in the current study, colloid osmotic pressure—primarily regulated by serum albumin—is a critical factor governing transvascular fluid movement and maintaining vascular–interstitial fluid balance. In the context of acute hemodilution, alterations in plasma protein levels can influence edema development by shifting the equilibrium between oncotic and hydrostatic pressures. Thus, incorporating oncotic parameters into the assessment of tissue fluid responses may enhance the overall understanding of fluid dynamics during hemodilution-related challenges [45]. Such observations are particularly pertinent in clinical settings involving patients with limited cardiopulmonary or metabolic reserves. It is important to note that measurements at a single time point following ANH may not adequately capture the clinical scenario. However, previous studies have indicated that tissue injury markers, including oxidative stress, edema, and cytokine expression, frequently peak one day post-ANH [37,46]. Therefore, obtaining measurements within the 24 h post-ANH period is crucial, and this provides the rationale for performing tissue analyses at the T2 time point in this study.

Although no significant intergroup differences were detected in most blood electrolytes and metabolites (Table 2), several trends in the ANH group suggest early physiological disturbance. The mild increase in sodium levels, despite remaining within physiological limits, likely reflects crystalloid-induced plasma dilution and compensatory tonicity regulation [47]. The increase in potassium levels observed at T_2_ can be attributed to several factors, including renal adaptive mechanisms, minor cellular leakage due to tissue stress, or possibly hemolysis associated with blood collection or reinfusion [43,44,48]. Furthermore, mild increases in calcium and decreases in glucose levels could reflect altered metabolic activity under hemodilution-induced stress [43,44]. Slight elevations in potassium and calcium may also indicate subtle renal modulation or cellular stress, potentially linked to apoptosis-related marker expression in renal and myocardial tissues [43,48]. The tendency toward lower glucose levels could reflect increased metabolic consumption or inflammatory activation, consistent with NF-κB and TNF-α pathway engagement during surgical stress [49,50]. Despite remaining within reference ranges, these deviations—combined with tissue-specific alterations in aquaporin—point to underlying organ-level responses beyond macrocirculatory endpoints [19,51]. Notably, the persistence of these metabolic shifts at the 24 h (T_2_) time point highlights that a single ANH event may lead to prolonged systemic stress, underscoring the relevance of extending experimental monitoring windows and integrating biochemical markers into perioperative fluid management strategies. The findings provide the rationale for performing tissue analyses at the T_2_ time point in this study.

Aquaporins (AQPs) are a major family of diverse transmembrane proteins. As essential cell water transporters, AQPs regulate osmoregulation and body water homeostasis. Their expression and types vary depending on the tissue and even the cell [9,52]. As shown in Figure 3, AQP1, AQP3, and AQP4 expression levels had different expression profiles in the aorta, heart, kidney, and liver tissues. The literature supports the finding that the AQP1 level was highest in the heart; as the most important aquaporin for this tissue, it has a critical function in maintaining cardiovascular homeostasis [16]. There was no significant change in AQP4 levels in the heart and aorta tissues. Notably, AQP1 levels decreased in the heart after ANH but significantly increased in the aortic tissue. Conversely, AQP3 levels were raised in the heart and reduced in the aorta due to ANH (Figure 4). These opposite responses in AQP1 and AQP3 suggested AQP translocation in the presence of a stimulant [53]; the regulatory functions of these AQPs are the subject of further research. AQP3 and AQP4 expressions were highest in kidney tissue; additionally, the AQP1 blot showed a high signal but was lower than that of the heart and liver (Figure 3). As primary regulators of water and salt metabolism, the kidneys express many AQPs, and AQP1–4 expressions are primarily associated with renal function [54]. While the AQP3 levels did not markedly change, the increase in AQP1 and decrease in AQP4 were significant (Figure 4). Additionally, AQPs have been associated with various hepatobiliary disorders, and AQP1 and AQP4 are involved in mechanisms of reabsorption/secretion of water [55,56], but there was only a significant increase in AQP3 levels in liver tissue (Figure 4).

Numerous reports have revealed a close relationship between AQP expression and injury caused by various stimulants [57]. Notably, as an inductor, hemodilution causes tissue damage with side effects such as edema, inflammation, and reduced regional oxygen delivery [36]; but the roles of AQPs in this process and their responses to the changing environment remain unclear. According to the data obtained in our study, ANH-induced apoptotic (Figure 5) and inflammatory (Figure 6) responses were detected in all of the tested tissues (the aorta, heart, liver, and kidney), supported by limited studies showing ANH-induced injury. Frazilio et al. [58] reported that neuronal apoptosis in the porcine hippocampus and cerebral cortex did not increase significantly in the acute phase of ANH, but hypoxia caused neuronal stress. The lack of apoptosis induction may be due to differences in the ANH model and the decrease in percentage HCT levels. In support of this, ANH in pigs has been associated with renal tissue edema, impaired microvascular oxygenation, and functional loss [37]. Furthermore, Lv et al. [59] reported increased NF-κB p65 and TNF-α levels in the cerebral cortex due to hemodilution. Moreover, increased AQP4 levels accompanying cerebral damage were reported in a porcine model after fluid resuscitation in hemorrhagic shock [11]. Additionally, AQP1 was associated with filtration in a fetal anemia model [12] and pulmonary edema in a newborn lamb model replicating infant CPB with hypothermic circulatory arrest [13]. Our correlation analysis between AQP levels and apoptotic (Figure 7) and inflammatory (Figure 8) responses showed tissue-specific AQP expression profiles. While no correlation was observed when all tissue results were evaluated together, remarkable relationships were detected when each tissue was assessed separately. Remarkably, AQP1, the most expressed aquaporin in the heart, was negatively correlated with damage-related inflammation markers NF-κB-p65 (master regulatory and manager transcription factor of inflammation) and TNF-α (a critical pro-inflammatory cytokine), while AQP3 was positively correlated with both apoptosis and inflammatory responses. On the contrary, AQP1 was positive, and AQP3 was inversely correlated in aorta samples, supporting immunoblot responses showing protein levels. This finding suggested that the decrease in AQP1 levels in the heart may be related to damage and that AQP1 may have been secreted and translocated into the aorta [53]. Additionally, a moderate inverse correlation was observed between AQP1 and TNF-α in the liver. Notably, an inverse correlation was observed between AQP4 levels and inflammation/apoptosis in kidney tissue, suggesting a protective role for AQP4 in this tissue.

## 5. Conclusions

This study provides the first evidence linking tissue-specific aquaporin (AQP1, AQP3, and AQP4) expression with apoptotic and inflammatory responses following hemodilution (in the ANH rat model). Nevertheless, this report was subject to certain limitations. The primary constraint was its reliance on short-term observation (specifically at the 24 h time point); thus, extended follow-up periods would provide a more comprehensive assessment of recovery or resolution. The use of whole-organ Western blot analysis (limitation: absence of cell-type specificity), and the lack of measurement of plasma protein concentrations, particularly albumin (limitation: inability to assess colloid osmotic pressure changes) were notable limitations. Despite its limitations, this study demonstrated that ANH produces both systemic and tissue-specific acute effects. Specifically, ANH altered the expression profiles of AQP1, AQP3, and AQP4 in a tissue-specific manner; the results suggest that AQP1 and AQP4 may play protective roles in the heart and kidney, respectively. In parallel, ANH induced transient yet clinically relevant systemic alterations, including elevated potassium, calcium, and lactate, as well as decreased glucose, pH, base excess, and PaCO_2_, indicating mild metabolic acidosis likely due to tissue hypoxia and impaired oxygen delivery. Notably, the persistence of metabolic deviations at 24 h post-intervention highlights the need for extended postoperative monitoring and the integration of molecular and biochemical indicators into fluid management strategies. These findings highlight the need for functional studies such as gene silencing or overexpression (limitation: lack of mechanistic validation) and serum-based AQP measurements (limitation: no evaluation of circulating AQP levels) to clarify the role of aquaporins in ANH-induced tissue stress. This research supports the development of AQP-targeted therapies, prompting further efforts to create AQP-specific treatments that mitigate ANH’s adverse effects.

## Figures and Tables

**Table 1:** Antibodies used for Western blotting.

Antibody	Host	Dilution	Catalog Number	Company *
**Anti-AQP1**	Rabbit	1:1000	AF5231	Affinity Biosciences
**Anti-AQP3**	Rabbit	1:1000	AF5222	Affinity Biosciences
**Anti-AQP4**	Rabbit	1:500	sc-20812	Santa Cruz Biotech.
**Anti-Bax**	Mouse	1:1000	NBP1-28566	Novus Bio.
**Anti-Bcl-2**	Rabbit	1:1000	NB100-56098	Novus Bio.
**Anti-Cas3^pro&cleaved^**	Rabbit	1:1000	AF6879	Affinity Biosciences
**Anti-NF-κB p65**	Rabbit	1:1000	A00284-1	Boster Bio. Tech.
**Anti-TNF-α**	Rabbit	1:1000	NB600-587	Novus Bio.
**Anti-β-actin**	Mouse	1:5000	MA5-15739	Thermo/Invitrogen
**Anti-Mouse IgG**	Goat	1:5000	31430	Thermo/Invitrogen
**Anti-Rabbit IgG**	Goat	1:5000	31460	Thermo/Invitrogen

* Affinity Biosciences (Liyang, China), Santa Cruz Biotechnology (Dallas, TX, USA), Boster Biological Technology (Valley Ave, Pleasanton, CA, USA), Novus Biologicals (St. Louis, MO, USA), Thermo/Invitrogen (Carlsbad, CA, USA).

**Table 2:** Demographic data and blood electrolyte/metabolite results.

		Control	Sham	ANH	
		Min–Max (Median) Mean ± SD	Min–Max (Median) Mean ± SD	Min–Max (Median) Mean ± SD	*p* Value
**Weight (g)**		400–500 (460)	405–510 (455)	420–500 (445)	0.9588
		455.00 ± 40.87	457.50 ± 38.44	455.00 ± 32.71	
**Estimated blood volume (mL)**		25.60–32 (27.84)	25.92–32.64 (30.40)	26.90–32 (28.50)	0.4641
		28.59 ± 2.55	30.03 ± 2.50	29.13 ± 2.10	
**Collected blood volume (mL)**		-	-	10.76–12.80 (11.46)	-
				11.67 ± 0.83	
**Blood Electrolytes and Metabolites**					
**Na** **(mmol/L)**	**T_BV_**	133–145 (139.5)	134–150 (141.5)	135–142 (139)	0.4168
		139.50 ± 4.68	142.30 ± 6.59	138.67 ± 2.50	
	**T_2_**	135–143 (137.5)	129–145 (138.5)	137–145 (141)	0.3351
		138.30 ± 3.45	137.30 ± 5.61	141.00 ± 3.41	
***p* Value**		0.6335	0.1875	0.2061	
**K** **(mmol/L)**	**T_BV_**	3.90–5.20 (4.20)	3.50–4.60 (4.20)	3.40–4.50 (4.00)	0.3039
		4.33 ± 0.54	4.15 ± 0.41	4.02 ± 0.42	
	**T_2_**	4.00–5.00 (4.35)	3.70–4.80 (4.40)	4.20–5.60 (4.85)	0.1297
		4.40 ± 40.87	4.35 ± 0.41	4.83 ± 0.50	
***p* Value**		0.9011	0.4164	0.0121 *	
**Ca (mmol/L)**	**T_BV_**	0.90–1.35 (1.18) 1.16 ± 0.16	1.25–1.32 (1.30) 1.29 ± 0.02	1.15–1.30 (1.20) 1.22 ± 0.06	0.1139
	**T_2_**	0.80–1.50 (1.05) 1.16 ± 0.16	1.08–1.38 (1.19) 1.20 ± 0.12	1.20–1.45 (1.30) 1.31 ± 0.10	0.1827
***p* Value**		0.7263	0.1068	0.1050	
**Cl** **(mmol/L)**	**T_BV_**	98–110 (101) 102.00 ± 4.29	101–107 (103) 103.30 ± 2.07	98–106 (101.5) 102.00 ± 3.03	0.7207
	**T_2_**	97–105 (102) 101.30 ± 2.94	99–104 (101.5) 101.70 ± 2.07	95–108 (103.5) 102.20 ± 4.88	0.9178
***p* Value**		0.7601	0.1925	0.9447	
**Glucose** **(mg/dL)**	**T_BV_**	99–203 (129) 142.30 ± 42.18	114–181 (138) 140.70 ± 23.17	102–185 (141) 140.80 ± 33.68	0.9956
	**T_2_**	117–185 (137) 143.80 ± 25.34	124–205 (134.5) 148.00 ± 30.15	102–204 (105) 136.30 ± 50.14	0.8580
***p* Value**		0.9419	0.6468	0.8588	

* *p* < 0.05 indicates comparison between T_BV_ and T_2_.

## Data Availability

Data available on request from the authors.

## Supplementary Materials

The following supporting information can be downloaded at https://www.mdpi.com/article/10.3390/medicina61091506/s1: Figure S1: Western blot uncropped images.

## Abbreviations

ANH	Acute normovolemic hemodilution
AQP	Aquaporin
Bax	Bcl-2-associated X protein
Bcl-2	B-cell lymphoma 2
Cas3	Caspase-3
CPB	Cardiopulmonary bypass
HCT	Hematocrit
NF-κB	Nuclear factor kappa B
PaCO_2_	Partial carbon dioxide pressure
TNF-α	Tumor necrosis factor alpha
