# Supplementary figures and images for: Acute Normovolemic Hemodilution Changes the Aquaporin Expression Profile in Specific Tissues and Induces Apoptotic and Inflammatory Processes in a Rat Model
Source: Medicina (Kaunas). 2025 Aug 22;61(9):1506. doi: 10.3390/medicina61091506 (PMC12471857; doi:10.3390/medicina61091506)

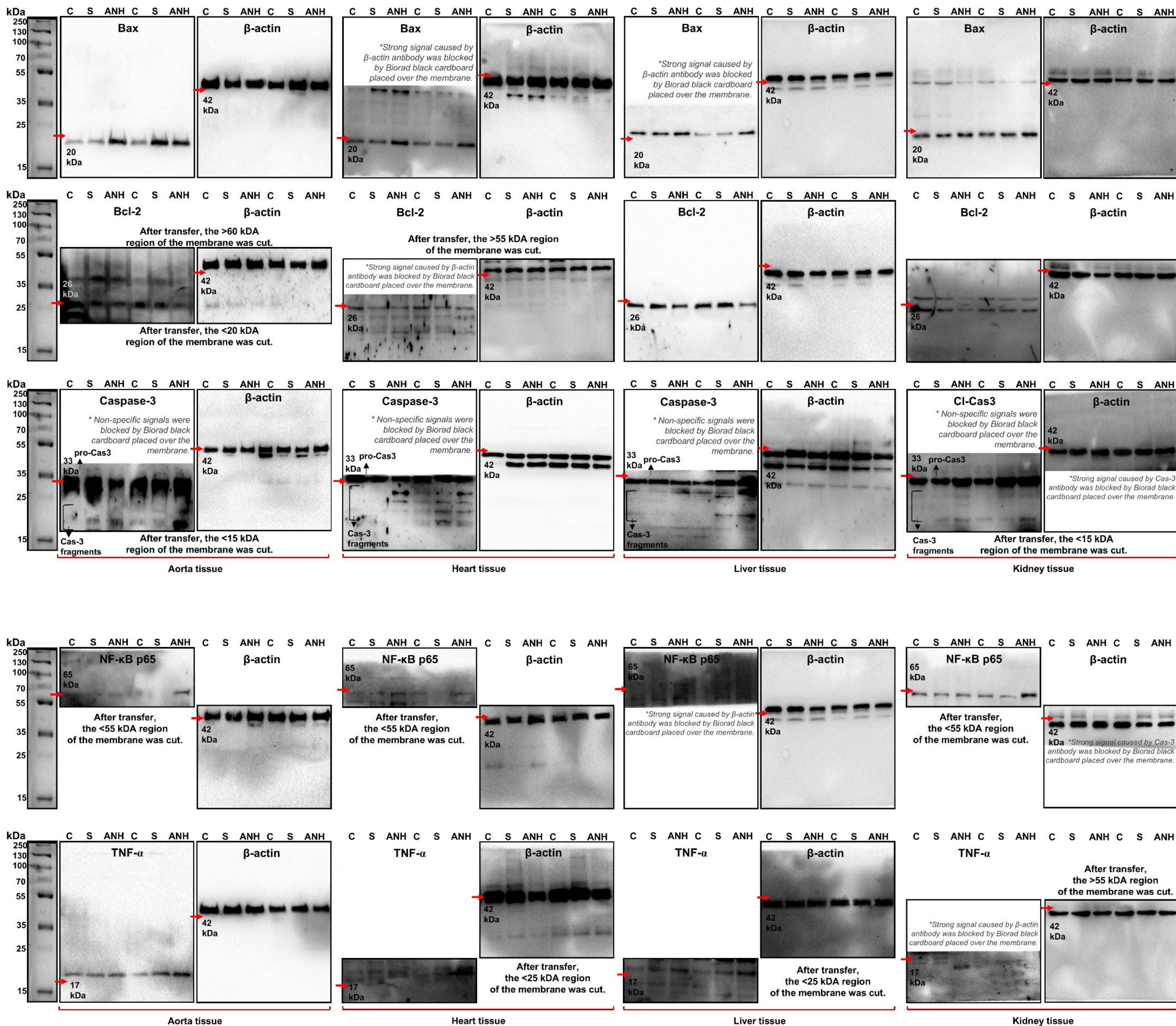

Supplement: Supplementary file 1 [file medicina-61-01506-s001.zip › medicina-3765264-supplementary.pdf]
